# Impact of vitamin D deficiency on maternal and birth outcomes in the Saudi population: a cross-sectional study

**DOI:** 10.1186/s12884-016-0901-4

**Published:** 2016-05-24

**Authors:** Ghadeer K. Al-Shaikh, Gehan H. Ibrahim, Amel A. Fayed, Hazem Al-Mandeel

**Affiliations:** Obstetrics and Gynecology Department, College of Medicine, King Khalid University Hospital, King Saud University, Riyadh, Kingdom of Saudi Arabia; College of Medicine, Princess Nourah Bint Abdulrahman University, Riyadh, Kingdom of Saudi Arabia; Department of Medical Biochemistry, Faculty of Medicine, Suez Canal University, Round Road, Ismailia, 41511 Egypt; Department of Biostatistics, High Institute of Public Health, Alexandria University, Alexandria, Egypt

**Keywords:** Gestational diabetes, Pregnancy, Vitamin D, Hypovitaminosis D, Adverse pregnancy outcome

## Abstract

**Background:**

Low serum vitamin D [25(OH)D] has been associated with different health problems worldwide. However, its causal role in several diseases remains unclear. We aimed to correlate vitamin D status with maternal and neonatal outcomes in pregnant females.

**Method:**

One thousand pregnant women were recruited during early labour from the labour ward of King Khaled University Hospital, Riyadh, Saudi Arabia. Detailed medical data of all participants were collected from their records. Delivery events and birth outcomes were also documented. Serum 25(OH)D levels were measured using an enzyme-linked immunosorbent assay. A receiver operating characteristic (ROC) curve was constructed to evaluate the ability of vitamin D levels to predict complicated pregnancies. Regression analysis was used to test the correlation between serum 25(OH)D levels and different variables.

**Results:**

Most of the participants were Saudis (89.9 %) and housewives (85.1 %) and 86.4 % of them had vitamin D deficiency (mean: 30.46 ± 19.6 nmol/L). Gestational diabetes mellitus (GDM) was the commonest complication detected (11.1 %) while the history of miscarriage was elevated (24.5 %). There was no association between GDM and low 25(OH)D. Yet, there was a significant negative correlation between serum 25(OH)D levels and fasting blood glucose among females older than 35 years (*r* = −0.2, *p* = 0.03). Hypertensive disorders of pregnancy and pre-existing hypertension were less than 1.5 % of frequency. Nevertheless, they were only recorded in women with insufficient and deficient vitamin D. ROC curve revealed that 25(OH)D levels were not able to discriminate between normal and adverse pregnancy outcomes (AUROC curve: 0.51; 95 % confidence interval (CI): 0.44–0.58; *p* = 0.8).

**Conclusion:**

Hypovitaminosis D, a highly prevalent health problem among pregnant females in Riyadh, has no relation to adverse pregnancy outcomes except for a higher prevalence of miscarriage in women with low 25(OH)D.

**Electronic supplementary material:**

The online version of this article (doi:10.1186/s12884-016-0901-4) contains supplementary material, which is available to authorized users.

## Background

Vitamin D, also known as calciferol, is a prohormone that plays an important role in calcium homeostasis and bone health in addition to its neuromuscular functions [1]. Vitamin D has two major forms, vitamin D_2_ and vitamin D_3_. They are further metabolized into 25-hydroxyvitamin D (25(OH)D) and 1,25- dihydroxy vitamin D (1,25(OH)2D), the active form of vitamin D, in the liver and the kidney, respectively [2, 3]. 25(OH)D is the main circulating vitamin D metabolite and its serum level serves as a biomarker of the vitamin status [4].

Beyond its effect on bone homeostasis, 25(OH)D has been implicated in varying clinical conditions. In the last two decades, the non-classical function of vitamin D has been suggested; it regulates a large number of human genes (~200 genes), resulting in a wide range of autocrine effects in different tissues [5]. For example, vitamin D is involved in regulation of cell proliferation, cell differentiation, and apoptosis [4]. In addition, it exerts immune responses through regulation of the innate and adaptive immunity [6]. This explains the correlation of hypovitaminosis D to the potential risk of a series of conditions like hypertension, diabetes mellitus, cancer, multiple sclerosis, allergy, asthma, autoimmune and infectious diseases as well as depression [7, 8].

In Saudi Arabia, hypovitaminosis D can be considered a major public health problem with a significantly high prevalence especially among women, ranging from to ~80 to 100 % in different studies [9–11]. Vitamin D deficiency can be seen not only in infancy and childhood but also across the other life stages from adolescence, adulthood, until old age [12]. The risk of vitamin D deficiency increases during pregnancy due to the increase in maternal and fetal demands [13]. Moreover, vitamin D is postulated to have a potential effect on several pregnancy outcomes including fetal skeletal outcome, hypertensive disorders, and gestational diabetes mellitus (GDM) [14–16]. To our knowledge, there is no published data on the relation of low vitamin D and pregnancy, or fetal development, in the Saudi population. The current study aimed to assess 25(OH)D levels in pregnant females residing in Riyadh, and to correlate the vitamin status to the possible maternal and neonatal adverse outcomes.

## Methods

This is a cross-sectional study where all consecutive pregnant females admitted in labour ward were invited to join the study. The study was conducted in accordance with the guidelines of the Declaration of Helsinki and was approved by the Institutional Review Board (IRB) of King Saud University. All pertinent study information was explained to all participants and they were informed that rejection or withdrawal from the study will not affect any medical service provided. A summarized study information sheet was shown to all cases before obtaining their verbal agreement. Finally, an informed verbal consent was obtained and witnessed by the attending nurse. A log book was created including the participant’s study number and the date of consent. The IRB waived the requirement for taking a written consent as the research had minimal risk of harm to subjects and involved no risky procedures for which written consent is required. The study was adherent to the STROBE criteria as outlined in Additional file 1.

### Inclusion and exclusion criteria

Recruitment of 1000 consecutive women was accomplished in the labour ward in King Khaled University Hospital (KKUH), Riyadh, Saudi Arabia, between the beginning of January till the end of June 2014. Exclusion criteria included known chronic illnesses [except hypertension and diabetes mellitus], metabolic bone disease, intestinal malabsorption, any evidence of liver, kidney, or gastrointestinal diseases), and the use of vitamin D supplements and/or other medications that affect vitamin D level (e.g. anticonvulsants, antituberculosis drugs).

### Data collection

Data on socio-demographics, medical and reproductive history were collected from all subjects. Medical records were abstracted to ascertain their anthropomorphic characteristics as well as their medical status throughout gestation. Participants’ pre-pregnancy body weights were not available in the record; pregnancy body mass index (BMI) was calculated using the admission weight and height measurements. All participants were screened for GDM by estimation of 75-g oral glucose tolerance test (OGTT) between 24 and 28 weeks of gestation. Data of OGTT and adverse pregnancy outcomes (e.g. GDM, gestational hypertension, preeclampsia and intrauterine growth restriction based on two measurements, at least, 2 weeks apart) were retrieved from their files. Diagnosis of preeclampsia was based on the new onset of hypertension (systolic blood pressure ≥140 mm Hg or diastolic blood pressure ≥90 mm Hg) after 20 weeks gestation in addition to proteinuria (≥300 mg/24 h). Gestational hypertension was defined as *De novo* hypertension alone, occurring after 20 weeks gestation in a previously normotensive woman [17]. Delivery events (e.g. preterm delivery, caesarean section) and birth outcomes (e.g. anthropometric birth outcomes, APGAR score and neonatal admission to the ICU) were recorded after labour.

### Estimation of serum 25(OH)D level

Blood samples were collected for vitamin D estimation, and sera were processed and stored at −80 °C until analyzed. Quantification of serum 25(OH)D was performed using a commercial enzyme-linked immunosorbent assay (ELISA) (K2110, Immunodiagnostic [Dutch Company], Holland). The range of detection was 5–300 nmol/L. Patients’ vitamin D status was evaluated according to the 25(OH)D concentrations into deficient [Levels below 50 nmol/L (20 ng/ml)], insufficient [concentrations between 51 and 74 nmol/L (21–29 ng/ml)] and normal [25(OH)D ≥75 nmol/L (≥30 ng/ml)] [18, 19].

### Statistical analysis

Sample size calculation was based on previous literature findings, where ~ 83 % of GDM patients suffered from vitamin D deficiency/insufficiency compared to 71 % in non-GDM subjects [20]. Assuming a significance level of 95 % (α = 0.05) and a power of 80 % (β = 0.20), calculation of the sample size revealed that the minimum number of participants required to reject the null hypothesis was 900. Statistical analysis was performed using the SPSS software v.20.0 for Windows® (SPSS Inc., Chicago, IL, USA). Data were presented as mean ± SD and percentages. Univariate analysis and differences between groups were assessed using the Student’s *t*-test, or Chi-square (*χ*^2^) test when appropriate. Receiver operating characteristic (ROC) curve was constructed and the area under the curve (AUC) was calculated to evaluate the specificity and sensitivity of vitamin D levels to predict complicated pregnancies from normal ones. Pearson correlation coefficient was used to test the correlation between quantitative variables. Linear regression statistics were used to determine the relationship between fasting OGTT and serum 25(OH)D levels with adjustment for age, BMI and parity. In addition, logistic regression analysis was adopted to assess the correlation between the history of miscarriage and the vitamin D levels adjusted for age and BMI. The values of 25(OH)D were log-transformed to achieve normality. All statistical tests were two-tailed, and a *p*-value <0.05 was considered statistically significant.

## Results

The age of women included in the study ranged from 17 to 47 years and ~ half of them were between 25 and 35 years. Most of the participants were Saudis (89.9 %) and housewives (85.1 %). Table 1 shows the socio-demographic data, reproductive information and outcomes of the current pregnancy in the studied population. The major form of pregnancy-associated complications was GDM (11.1 %), followed by intrauterine growth restriction and gestational hypertension (1.5 and 1.4 %, respectively) and the least one was preeclampsia (0.9 %). Out of the 19 patients (1.9 %) who had pre-gestational diabetes 14 had type-1 diabetes mellitus (1.4 %) and five had type-2 (0.5 %). Surprisingly, history of miscarriage among pregnant females was highly elevated (24.5 %). According to the pregnancy BMI, 56.7 % of the women were higher than 30 kg/m^2^, while 31 % ranged between 25 and 30 kg/m^2^ and 12.3 % was below 25 kg/m^2^.

**Table 1:** Socio-demographic and clinical data of the study population

Age (years; mean ± SD [range])	29.03 ± 5.7 [17–47]
Age distribution^a^	
Less than 25 years	321 (32.2)
25–35 years	513 (51.4)
More than 35	164 (16.4)
Nationality	
Saudi	899 (89.9)
Non Saudi	101 (10.1)
Education^a^	
High school	668 (70.4)
University or higher	281 (29.6)
Working status^a^	
Housewife	851 (85.3)
Employee	147 (14.7)
Reproductive history	
Parity	
Primiparous	12 (31.6)
Multiparous	26 (68.4)
Positive history of multiple pregnancies	56 (5.6)
Positive history of miscarriage	245 (24.5)
Status of current pregnancy	
Smoking during pregnancy^a^	20 (0.2)
Gestational age till delivery (Weeks; mean ± SD [range])	38.7 ± 1.9 [23–42]
Pregnancy BMI (Kg/m^2^; mean ± SD [range])	30.9 ± 6.7 [14.5–55.8]
Gestational diabetes	111 (11.1)
Pre-Gestational diabetes	19 (1.9)
Pre-existing hypertension	4 (0.4)
Gestational hypertension	14 (1.4)
Preeclampsia	9 (0.9)
Intrauterine growth restriction	15 (1.5)
Delivery and neonatal outcomes	
Preterm labour	80 (8.0)
Induction of labour	128 (12.8)
Mode of delivery	
Spontaneous	768 (76.8)
Instrumental delivery	61 (6.1)
Caesarian Section	171 (17.1)
Baby’s weight (Kg; mean ± SD [range])	3.1 ± 0.5 [1.06–5.3]
Baby’s length (cm; mean ± SD [range])	49.5 ± 2.7 [34–62]
Baby’s head circumference (cm; mean ± SD [range])	34.1 ± 1.9 [26–52]
APGAR score	8.6 ± 0.63 [7–9]
Neonatal admission to ICU	29 (2.9)
Vitamin D level (nmol/L; mean ± SD [range])	30.46 ± 19.6 [7.1–150]

^a^Total number is below one thousand due to incomplete questionnaires; data are expressed in number (percentage) unless specified; *SD* Standard deviation

Serum 25(OH)D levels ranged from 7.1 to 150 nmol/L. Division of the participants according to their vitamin D status revealed that the majority of women had vitamin D deficiency (86.4 %; mean: 24.2 ± 10.6 nmol/L; range: 7.1–49.9). Table 2 shows a comparison between study participants’ data, pregnancy and birth outcomes in relation to their vitamin D status. Women with deficiency were mostly in the middle age group [25–35 years] and they were housewives (*p* < 0.01 and = 0.02, respectively). There was no difference in the GDM frequencies in vitamin D status subgroups. Moreover, there was a significant increase in the percentage of women with positive history of miscarriage in the insufficient status compared to the deficient and normal ones (*p* = 0.02) (Table 2). On the other hand, gestational hypertension, pre-existing hypertension, preeclampsia and pre-existing diabetes mellitus were only recorded in women with deficient and insufficient vitamin D. However, these frequencies did not reach significant levels (Table 2). Furthermore, there was no difference between women with low vitamin D and those with normal vitamin status regarding birth outcomes (*p* > 0.05).

**Table 2:** Comparison of participants’ data, pregnancy and neonatal outcomes according to their vitamin D status

	Normal vitamin D *N* = 38	Insufficient vitamin D *N* = 98	Deficient vitamin D *N* = 864	*P* value
Age groups distribution				
Less than 25 years	11 (28.9)	20 (20.4)	290 (33.6)	<0.01
25–35 years	13 (34.2)	52 (53.1)	448 (52.0)	
More than 35	14 (36.8)	26 (26.5)	124 (14.4)	
Nationality				
Saudi	32 (84.2)	85 (86.7)	782 (90.5)	0.24
Non Saudi	6 (15.8)	13 (13.3)	82 (9.5)	
Education				
High school	22 (61.1)	62 (65.3)	584 (71.4)	0.32
University or higher	14 (38.9)	33 (34.7)	234 (28.6)	
Working status				
Housewife	28 (73.7)	78 (79.6)	745 (86.4)	0.02
Employee	10 (26.3)	20 (20.4)	117 (13.6)	
Reproductive history				
Parity				
Primiparous	12 (31.6)	24 (24.5)	287 (32.2)	0.22
Multiparous	26 (68.4)	74 (75.5)	577 (66.8)	
Positive history of miscarriage	7 (18.4)	35 (35.7)	203 (23.5)	0.02
Status of current pregnancy				
Pregnancy BMI (Kg/m^2^; mean ± SD)	30.7 ± 5.5	30.7 ± 6.2	31.1 ± 6.9	0.66
Gestational diabetes	5 (13.5)	12 (13.3)	94 (12.0)	0.8
Pre-Gestational Diabetes	0 (0.0)	1 (1.1)	18 (2.3)	0.9
Pre-existing hypertension	0 (0.0)	1 (1.0)	3 (0.3)	0.44
Gestational hypertension	0 (0.0)	2 (2.0)	12 (1.4)	0.79
Preeclampsia	0 (0.0)	0 (0.0)	9 (1.0)	0.28
Intrauterine growth restriction	2 (5.3)	2 (2.1)	11 (1.3)	0.07
Delivery and neonatal outcomes				
Preterm labour	4 (10.8)	3 (3.1)	73 (8.5)	0.14
Mode of Delivery				
Spontaneous	29 (76.3)	76 (77.6)	663 (76.7)	
Instrumental delivery	1 (2.6)	4 (4.1)	56 (6.5)	0.71
Caesarian section	8 (21.1)	18 (18.4)	145 (16.8)	
Birth weight (Kg; mean ± SD)	3.1 ± 0.5	3.2 ± 0.4	3.1 ± 0.5	0.67
Baby’s length (cm; mean ± SD)	49.5 ± 2.2	49.7 ± 2.2	49.5 ± 2.8	0.96
Head Circumference (cm; mean ± SD)	34.1 ± 1.1	34.4 ± 1.7	34.1 ± 2.2	0.6
APGAR score (mean ± SD)	8.3 ± 1.9	8.9 ± 0.3	8.8 ± 0.8	0.05
Neonatal admission to ICU	1 (2.6)	0 (0.0)	28 (3.3)	0.18

Data are expressed in number (percentage) unless specified; *SD* Standard deviation

**Fig. 1:**
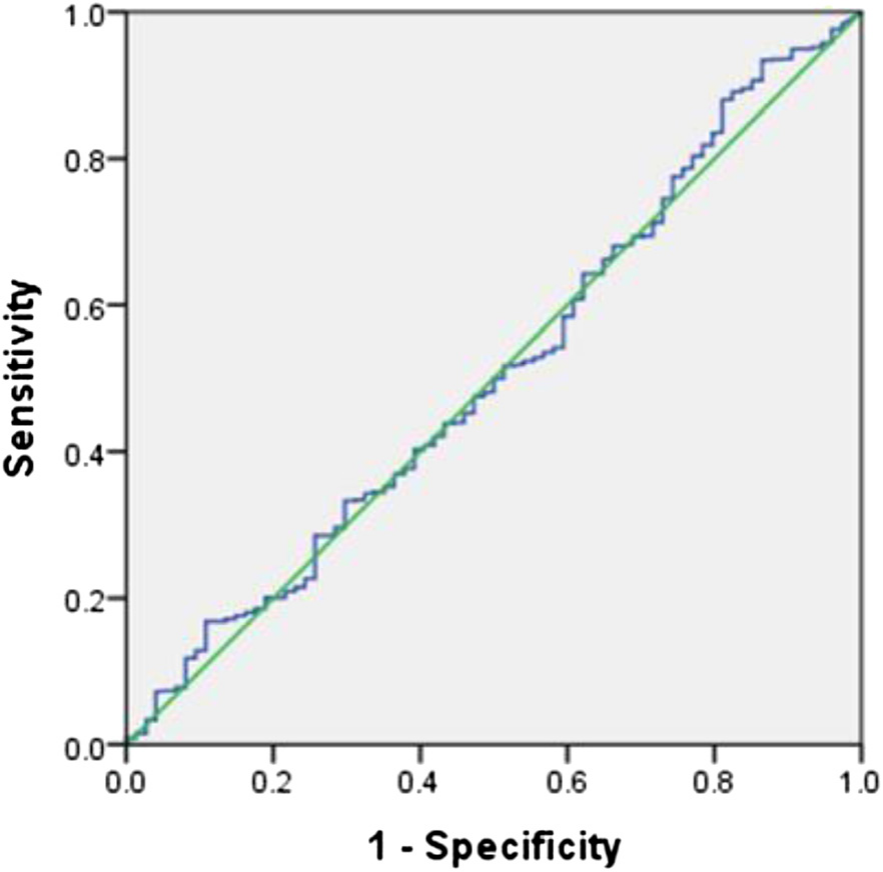
ROC curve analysis of serum Vitamin D and its relation to normal pregnancy outcome discrimination. AUROC curve: 0.51; 95 % confidence interval (CI): 0.44–0.58; *p* = 0.8

**Fig. 2:**
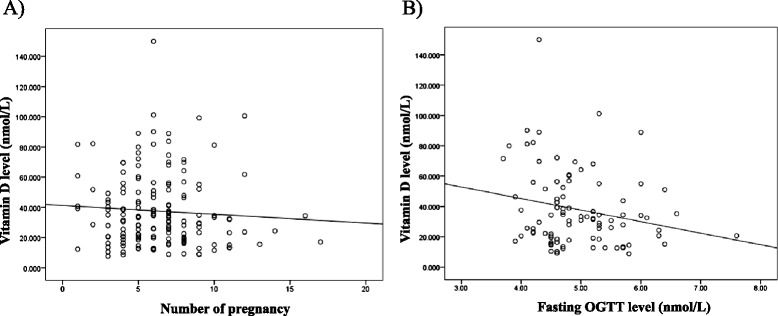
Correlation between vitamin D levels and pregnancy status. **a** A weak negative correlation between vitamin D levels and parity (*r* = −0.07, *p* = 0.3). **b** A significant negative correlation between the vitamin D levels and the fasting levels of oral glucose tolerance test (OGTT) among females aging more than 35-year-old (*r* = −0.2, *p* = 0.03)

Seventy-four percent of women had a normal pregnancy, delivery and neonatal outcomes. This sub-group of participants had a mean vitamin D level of 29.6 ± 18.9 nmol/L (range: 7.5–101). A ROC curve was plotted to investigate the potential ability of serum 25(OH)D to identify normal pregnancy outcomes (Fig. 1). Vitamin D levels were not able to discriminate between normal pregnancies and birth outcomes and any possible complication (AUROC curve: 0.51; 95 % confidence interval (CI): 0.44–0.58; *p* = 0.8). A noticed significant age discrepancy was confirmed between vitamin D subgroups. As there might be a co-linearity between age and parity, a stratified analysis of the correlation between 25(OH)D and parity among different age groups was conducted. A weak negative correlation between vitamin D levels and parity was apparent, especially in older age groups (>35 years) (*r* = −0.07, *p* = 0.3) (Fig. 2a). On the other hand, a significant negative correlation was evident between the levels of 25(OH)D and those of fasting OGTT among the oldest age group (>35 years) (*r* = −0.2, *p* = 0.03) (Fig. 2b). Adjustment for age, BMI and parity via linear regression model revealed a weak negative relationship between serum 25(OH)D and fasting OGGT levels; however, this did not reach statistical significance (β = −0.07, adjusted *r*^2^ = 0.04, *p* = 0.16). Moreover, logistic regression analysis showed that women with higher levels of vitamin D were less likely to report the history of miscarriage (odds ratio 0.7, 95 % confidence interval: 0.35–1.45, *p* = 0.3); yet, this correlation was not statistically significant.

## Discussion

The presence of vitamin D receptors (VDR) in almost every tissue drew the attention to the identification of the extra-skeletal functions of vitamin D [21]. So far, thousands of research have been published to explore the implication of vitamin D in human illness. It has also been correlated to maternal and fetal health during pregnancy [22]. Despite abundant sunlight, hypovitaminosis D is highly prevalent among the Saudi population. It is more frequent in the young and middle-aged group of apparently healthy Saudi adults [23] and in females more than males [10]. A limited number of publications in Saudi Arabia studied vitamin D deficiency in relation to diseases beyond bone health, e.g. diabetes mellitus [23, 24] and obesity [25]. Al-Mogbel [9] investigated vitamin D levels in Saudi females in the childbearing period and reported that all participants had hypovitaminosis D and the majority had a severe form of deficiency (~79 %). In spite of this high figure, no study has been published in the kingdom of Saudi Arabia about the effect of vitamin D deficiency on maternal and birth outcomes.

The current study revealed that vitamin D deficiency was highly prevalent, mostly in the middle-aged pregnant women. Overall, vitamin D status had no relation to the risk of adverse pregnancy and neonatal outcomes. 25(OH)D concentrations showed no association with the risk of GDM while hypertensive disorders of pregnancy existed only in women with insufficient and deficient vitamin D. In addition, positive history of miscarriage was highly elevated in women with vitamin D insufficiency.

GDM was the most common maternal complication in this study. Its prevalence was consistent with that reported globally (4.1–27.5 %) [26]. Previous reports associated vitamin D deficiency with GDM [27, 28] and in several countries like Iran [29], Australia [30] and the United States [16]. On the contrary and similar to our results, no significant association was reported by Rodriguez et al. [31] and Flood-Nichols et al. [32] and in different countries too, e.g. India [33], the United Kingdom [34] and the United States [35]. It should be noted that vitamin D supplementation during early pregnancy decreased the incidence of GDM in women having vitamin D level below 80 nmol/L, yet this frequency was not significant when compared to the one who did [36]. On the other hand, an association of vitamin D deficiency with impaired insulin secretion has been observed in different studies [37, 38]. Inadequate insulin secretion has been identified in rodents with vitamin D deficiency [39]. In addition, a significant correlation was described between 25(OH)D levels in pregnant women and insulin sensitivity or fasting blood glucose levels [40]. This might explain the weak negative correlation between fasting blood glucose and vitamin D levels in our study. A similar inverse correlation was also observed by Tsur et al. with more potency to the progression from normoglycemia to hyperglycemia in patients with severe vitamin D deficiency (levels less than 25 nmol/L) [41].

Preeclampsia, an adverse effects ranging from 2 to 17 % in pregnant women worldwide [42], has been diagnosed in less than 1 % of our study population. Though, hypertensive disorders of pregnancy existed only in pregnant women with insufficient and deficient vitamin D. Vitamin D and sunlight may have a role in the incidence of preeclampsia that is more frequent in winter than summer [43]. Nonetheless, controversy remains about the relationship of vitamin D deficiency with preeclampsia [44], and on the effectiveness of vitamin D supplementation in reducing its risk during pregnancy [45].

Apparently, low 25(OH)D concentrations were not associated with birth and neonatal adverse effects in our study. Despite the paucity of research studying pregnancy outcomes in relation to vitamin D status, several studies recorded findings similar to our results [31, 46, 47]. However, the high frequency of history of miscarriage identified in this research was associated mainly with vitamin D insufficiency. Previous studies attempted to examine the role of low vitamin D in complications like preterm delivery or infertility with no records on its relation to miscarriage. The high rate of history of miscarriage detected herein might be related to the proposed regulatory role of vitamin D ingenes associated with placental invasion, normal implantation, and angiogenesis [48]. In addition, vitamin D may play a potential role in the prevention of miscarriage due to its combined immunomodulatory and anti-inflammatory properties during early pregnancy [49]. Another factor is the high association of vitamin D deficiency with infectious diseases, specifically with bacterial vaginosis [50]. Whatever their types or sites, these infections represent a major risk to the maternal health and might lead to serious pregnancy adverse outcomes.

Pregnancy is a special condition during which the body experiences several physiological alterations, including changes in vitamin D metabolism. The effect of vitamin D deficiency on maternal and neonatal health is still under investigations. The controversy between the results of documented studies did not give obvious clues to the real association of hypovitaminosis D with maternal and neonatal complications [51]. So far, vitamin D supplementation during pregnancy had no effect on the incidence of adverse effects like preeclampsia, GDM, preterm birth, small-for-gestational-age infants, and cesarean section [45]. Nevertheless, these data were retrieved from trials conducted on populations living in the northern latitudes. It cannot be generalized to countries like Saudi Arabia, where there is enough sun exposure. Though sartorial parameters like the cultural practice of complete covering of the body, head and even face, in addition to the restriction of outdoors activities, might counteract this positive effect of the ample sunlight [24, 52, 53]. It is highly recommended putting into consideration each population characteristic while investigating the role of hypovitaminosis D in relation to pregnancy outcome. Factors such as geographic location and latitude, ethnicity, skin phenotype and individual response to UV, age, physical activity and poor diet have to be interpreted cautiously [8, 51, 53]. Even vitamin D supplementation should be adjusted in relation to the population reference baseline, especially in pregnant women residing in countries with high prevalence of hypovitaminosis D like Saudi Arabia [51].

Maternal vitamin D levels vary during gestation. Serum 1,25(OH)D increases normally from the end of the first trimester and reaches its maximum level in the third trimester [54]. However, associated increase in 25(OH)D levels could not be identified [55]. Moreover, Vitamin D action is affected by factors like its metabolism and other hormonal and metabolic pathways [56]. It can be speculated that factors other than vitamin D can determine maternal and neonatal outcomes. Furthermore, vitamin D action is also dependent on its interaction with its binding protein and its receptor [56]. In fact, genetic variations (e.g. Vitamin D receptor polymorphisms) can be involved in vitamin D metabolism and in disease susceptibility [57]. Such population differences could explain the disparity in data published regarding the effect of vitamin D on pregnancy outcomes. Finally, the increased oxidative stress associated with any placental dysfunction causes an alteration in the expression of vitamin D-binding protein and vitamin D receptors [58] that subsequently can alter the vitamin D action.

This study provided novel information about the relation of vitamin D status and pregnancy outcomes in the Saudi population. It was conducted on a large sample size, yet it had some limitations. Data of vitamin D concentrations during early pregnancy were not available as pregnant women were reluctant to follow up in governmental hospitals. Confounders like pre-pregnancy BMI, lifestyle and physical activity were not investigated. Our data revealed that vitamin D deficiency has no effect on the risk of adverse pregnancy and birth outcomes. However, the study design is of cross-sectional nature; this hindered the confirmation of the causal relationship between vitamin D levels and history of miscarriage, a common unfavorable pregnancy outcome reported in this research.

## Conclusion

The study underscores the importance of measurement of serum 25(OH)D within the Saudi population. Nevertheless, hypovitaminosis D showed no relation to adverse pregnancy outcomes in this research. Regardless of all associations or correlation studies that have been published, none can give a direct proof of the causality of vitamin D deficiency in different pregnancy outcomes. Further interventional and experimental studies must be conducted to clarify the exact implication of vitamin D in inducing these adverse effects. In addition, a prospective study is needed to give stronger evidence on the suggested correlation between vitamin D and miscarriage. Although there is a lack of agreement on the need for vitamin D intake during pregnancy, vitamin supplementation is still recommended until this dilemma is deciphered.

## Ethics and consent

The study was conducted in accordance with the guidelines of the Declaration of Helsinki and was approved by the Institutional Review Board (IRB) of King Saud University (Approval number: E-10-218).

All pertinent study information was explained to them and they were informed that rejection or withdrawal from the study will not affect any medical service provided. A summarized study information sheet was shown to all cases before obtaining their verbal agreement. Finally, an informed verbal consent was obtained and witnessed by the attending nurse. A log book was created including the participant’s study number and the date of consent. The IRB waived the requirement for taking a written consent as the research had minimal risk of harm to subjects and involved no risky procedures for which written consent is required.

## Additional files

Additional file 1: STROBE Statement. (DOC 92.5 kb)

## Abbreviations

1,25(OH)2D: 1,25-dihydroxy vitamin D
25(OH)D: 25-hydroxyvitamin D
AUC: area under the curve
BMI: body mass index
CI: confidence interval
ELISA: enzyme-linked immunosorbent assay
GDM: gestational diabetes mellitus
ICU: intensive care unit
IRB: Institutional Review Board
KKUH: King Khaled University Hospital
OGTT: oral glucose tolerance test
ROC: receiver operating characteristic
SD: standard deviation
VDR: vitamin D receptors
