# Supplementary material for: Impact of vitamin D deficiency on maternal and birth outcomes in the Saudi population: a cross-sectional study
Source: BMC Pregnancy Childbirth. 2016 May 24;16:119. doi: 10.1186/s12884-016-0901-4 (PMC4879737; doi:10.1186/s12884-016-0901-4)
Supplement: Additional file 1: — STROBE Statement. (DOC 92.5 kb) [file 12884_2016_901_MOESM1_ESM.doc]

STROBE Statement—checklist of items that should be included in reports of observational studies

|  | Item No | | Recommendation | Page Number of the points addressed in the manuscript | |  |
| --- | --- | --- | --- | --- | --- | --- |
| **Title and abstract** | 1 | | (*a*) Indicate the study’s design with a commonly used term in the title or the abstract | 1 | |  |
|  |  |  | (*b*) Provide in the abstract an informative and balanced summary of what was done and what was found | 3 | |  |
| Introduction | | | |  | |  |
| Background/rationale | 2 | | Explain the scientific background and rationale for the investigation being reported | 5, 6 | |  |
| Objectives | 3 | | State specific objectives, including any prespecified hypotheses | 6 | |  |
| Methods | | | |  | |  |
| Study design | 4 | | Present key elements of study design early in the paper | 6 | |  |
| Setting | 5 | | Describe the setting, locations, and relevant dates, including periods of recruitment, exposure, follow-up, and data collection | 6, 7 | |  |
| Participants | 6 | | (*a*) *Cross-sectional study*—Give the eligibility criteria, and the sources and methods of selection of participants | 6 | |  |
|  |  |  | (*b*) *Cohort study*—For matched studies, give matching criteria and number of exposed and unexposed  *Case-control study*—For matched studies, give matching criteria and the number of controls per case | N/A | |  |
| Variables | 7 | | Clearly define all outcomes, exposures, predictors, potential confounders, and effect modifiers. Give diagnostic criteria, if applicable | 7 | |  |
| Data sources/ measurement | 8* | | For each variable of interest, give sources of data and details of methods of assessment (measurement). Describe comparability of assessment methods if there is more than one group | 7 | |  |
| Bias | 9 | | Describe any efforts to address potential sources of bias | 7, 8 | |  |
| Study size | 10 | | Explain how the study size was arrived at | 8 | |  |
| Quantitative variables | 11 | | Explain how quantitative variables were handled in the analyses. If applicable, describe which groupings were chosen and why | 8 | |  |
| Statistical methods | 12 | | (*a*) Describe all statistical methods, including those used to control for confounding | 8 | |  |
|  |  |  | (*b*) Describe any methods used to examine subgroups and interactions | 8 | |  |
|  |  |  | (*c*) Explain how missing data were addressed |  | |  |
|  |  |  | (*d*) *Cross-sectional study*—If applicable, describe analytical methods taking account of sampling strategy | 6 | |  |
|  |  |  | (*e*) Describe any sensitivity analyses | 7 | |  |
| Results | | | | |  | |
| Participants | 13* | (a) Report numbers of individuals at each stage of study—eg numbers potentially eligible, examined for eligibility, confirmed eligible, included in the study, completing follow-up, and analysed | | | 6, 7 | |
|  |  | (b) Give reasons for non-participation at each stage | | | N/A | |
|  |  | (c) Consider use of a flow diagram | | | N/A | |
| Descriptive data | 14* | (a) Give characteristics of study participants (eg demographic, clinical, social) and information on exposures and potential confounders | | | 9, 25-28 | |
|  |  | (b) Indicate number of participants with missing data for each variable of interest | | | 25, 26 | |
|  |  | (c) *Cohort study*—Summarise follow-up time (eg, average and total amount) | | | N/A | |
| Outcome data | 15* | *Cohort study*—Report numbers of outcome events or summary measures over time | | | N/A | |
|  |  | *Case-control study—*Report numbers in each exposure category, or summary measures of exposure | | | N/A | |
|  |  | *Cross-sectional study—*Report numbers of outcome events or summary measures | | | 9, 10 | |
| Main results | 16 | (*a*) Give unadjusted estimates and, if applicable, confounder-adjusted estimates and their precision (eg, 95% confidence interval). Make clear which confounders were adjusted for and why they were included | | | 10 | |
|  |  | (*b*) Report category boundaries when continuous variables were categorized | | | - | |
|  |  | (*c*) If relevant, consider translating estimates of relative risk into absolute risk for a meaningful time period | | | - | |
| Other analyses | 17 | Report other analyses done—eg analyses of subgroups and interactions, and sensitivity analyses | | | 9, 10, 25-28, figure 1 and 2 | |
| Discussion | | | | |  | |
| Key results | 18 | Summarise key results with reference to study objectives | | | 11 | |
| Limitations | 19 | Discuss limitations of the study, taking into account sources of potential bias or imprecision. Discuss both direction and magnitude of any potential bias | | | 14 | |
| Interpretation | 20 | Give a cautious overall interpretation of results considering objectives, limitations, multiplicity of analyses, results from similar studies, and other relevant evidence | | | 11- 15 | |
| Generalisability | 21 | Discuss the generalisability (external validity) of the study results | | | 14, 15 | |
| Other information | | | | |  | |
| Funding | 22 | Give the source of funding and the role of the funders for the present study and, if applicable, for the original study on which the present article is based | | | 16 | |

*Give information separately for cases and controls in case-control studies and, if applicable, for exposed and unexposed groups in cohort and cross-sectional studies.
